# Analysis of the safety and efficacy of flow diverter device in the treatment of tandem aneurysms in the internal carotid artery

**DOI:** 10.3389/fneur.2025.1462108

**Published:** 2025-01-20

**Authors:** Jun Wan, Ligang Xu, Yeqing Jiang, Lei Zhang, Zhenyu Wang, Xiaolong Zhang, Shengzhang Wang

**Affiliations:** ^1^Academy for Engineering and Technology, Fudan University, Shanghai, China; ^2^Department of Interventional Radiology, Jing’an District Central Hospital, Fudan University, Shanghai, China; ^3^Department of Radiology, Huashan Hospital Affiliated to Fudan University, Shanghai, China; ^4^Institute of Biomechanics, Department of Aeronautics and Astronautics, Fudan University, Shanghai, China

**Keywords:** intracranial aneurysms, tandem aneurysms, flow diverter device, pipeline, Tubridge

## Abstract

**Objective:**

To investigate the clinical efficacy and safety of flow diverter device (FDD) in the treatment of tandem aneurysms in the internal carotid artery.

**Materials and methods:**

This study was a retrospectively observational study involving two neurointerventional centers. Sixteen Patients with tandem aneurysms in the internal carotid artery treated with FDD and Digital Subtraction Angiography (DSA) follow-up in Huashan Hospital Affiliated with Fudan University and Jing’an District Central Hospital Affiliated with Fudan University from 2020.08 to 2023.12 were included. The outcomes included the angiographic occlusion rate of aneurysms, complications, and the modified Rankin Scale score. The risk factors of complete occlusion of tandem aneurysms were explored by logistic regression analysis.

**Results:**

A total of 38 aneurysms were included, including 21 aneurysms of 8 patients in the Pipeline Embolization Device (PED) group and 17 aneurysms of 8 patients in the Tubridge Flow Diverter (TFD) group. A total of 16 FDD stents were implanted, 8 in each PED and TFD group, with a technical success rate of 100%. The median value of maximum aneurysm diameter (D_max_) was 4.27 (3.57–5.41) mm. Among them, 28 aneurysms had a maximum diameter of <5 mm (73.7%), 10 aneurysms had a maximum diameter of 5–15 mm (26.3%). All patients were followed up clinically for a median of 25.5 months (15.5–28.7 months). There were no deaths and symptomatic complications. The modified Rankin Scale scores (mRS) of 16 patients were all less than 2. All patients were examined by angiography with a median of 14 months (6–27 months). Among them, there were 5 Consensus Grading Scale for Endovascular Aneurysm Occlusion (CGSFEAO) grade 5 (13.2%), 1 CGSFEAO grade 4 (2.6%), 1 CGSFEAO grade 2 (2.6%) and 31 CGSFEAO grade 0 (81.6%). The complete occlusion rate of intracranial aneurysm in the whole study was 81.6%, including 85.7% in the PED group and 76.5% in the TFD group, and there was no significant statistical difference between the two groups. Statistically significant variables were not found in univariate logistic regression analysis.

**Conclusion:**

FDD is safe and effective in treating tandem aneurysms in the internal carotid artery with a high occlusion rate and few complications. TFD is comparable to PED in the treatment of intracranial tandem aneurysms.

## Introduction

Tandem aneurysms (TAs) are a special type of multiple intracranial aneurysms (IAs) defined as IAs near each other on the same parent artery or adjacent artery (1, 2). The incidence of TAs accounting for 1.7–4.2% is low, and the treatment strategy is inconclusive. Surgical clipping and endovascular intervention are the first-line treatment for Tas (1, 2). These lesions are often accompanied by segmental vascular abnormalities, coupling with their special positional relationship, making traditional treatment extremely challenging (3). As a new-emerging endovascular treatment, Flow diverter Devices (FDD) have been applied to various intracranial aneurysms containing small and medium-sized aneurysms, tandem aneurysms, and so on (4–7). Theoretically, it can treat multiple aneurysms and segmental vascular abnormalities in a single operation. It helps to simplify the operation and reduce the incidence of surgical complications and aneurysm recurrence (1, 8). However, there are few reports on FDD treating tandem aneurysms in the anterior circulation. Therefore, the clinical data of 16 consecutive patients with unruptured tandem aneurysms in the internal carotid artery admitted to two neuro-interventional centers were retrospectively analyzed, to explore the clinical efficacy of FDD in treating TAs.

## Materials and methods

### Population

All patients or their legal representative signed the informed consent for minimally invasive interventional surgery. Sixteen patients with unruptured intracranial tandem aneurysms in the internal carotid artery who implemented FDD in Huashan Hospital affiliated with Fudan University and Jing’an District Central Hospital affiliated with Fudan University were retrospectively included between August 2020 and December 2023. Pipeline Embolization Device (PED; covidien, Irvine, California) and Tubridge Flow Diverter (TFD; microPort NeuroTech, Shanghai, China) were included.

Inclusion criteria: (1) 18–80 years old; (2) >1 unruptured located adjacent to each other in the internal carotid artery (ICA); (3) Implementing PED/TFD, combining with coils or none.

Exclusion criteria: (1) Absence of Digital Subtraction Angiography (DSA) follow-up results. There were no follow-up of 3 cases in PED group and 1 case in TFD group.

### Endovascular treatment

All operations were performed under general anesthesia and heparinization, and the femoral artery approach was used. An 8F Neuron™ MAX guiding catheter (Genesis MedTech, Shanghai, China) or 8F MidAccess 088 Guide Catheter (Precision Medical, Nanjing, China) was inserted into the petrous segment of the internal carotid artery through a 5F 125cm MP A1 angiographic catheter (Cordis, Miami Lakes, America) and a 0.035inch guide wire (Terumo, Tokyo, Japan) to establish the pathway. If the blood vessels were extremely curved, a 5/6F Tethys intermediate guiding catheter (Achieva Medical, Jiangsu, China) could be applied to the cavernous sinus segment of the internal carotid artery for strengthening support. If a type III aortic arch was encountered, two guide wires (0.035 and 0.038 inches, Terumo, Tokyo, Japan) could be used to guide the guiding catheter into the internal carotid artery.

Firstly, the stent size was determined based on the maximum proximal diameter of the parent artery. Generally, the distal and proximal ends of the stent were placed in the flat segment by default. The DSA analysis tool (Philip, Netherlands) was used to measure the vessel diameter with the catheter as the scale, and the maximum diameter of the proximal vessel of the parent artery was measured from multiple angles. In special cases, the diameter difference between the distal and proximal segments of the parent artery was large, and the stent size was preferred to be larger. Secondly, the tangent position of the aneurysm and the distal and proximal straight segments of the parent artery were selected as the working Angle of stent release. Thirdly, stent delivery and release; Shaping the micro-guide wire at the end of the stent *in vitro* was the key element of intraoperative safety. The stent catheter (Fasttrack Catheter for TFD, MicroPort NeuroTech, Shanghai, China; Marksman Catheter for PED, ev3/Covidien, America) was delivered to the M2 segment of the middle cerebral artery, and then the stent was delivered in place. The position of the catheter and stent was adjusted to release the stent *in situ*. During the release process, attention should be paid to the relative position of the guide wire tip of the stent and the branch of the parent artery to avoid damage to the branch vessels. The stent was slowly released, pushed, and observed from multiple angles to confirm its apposition. Low tension was released at the end of the stent. Fourthly, the stent apposition was carefully observed. The microcatheter combined with “J” shaped micro-guide wire (Synchro^2^, Stryker, America) massaged the stent to make it adhere to the wall well, and the operation was slow and careful to avoid the compression and displacement of the stent. After multi-angle angiography, the relative position relationship between the stent and the vessel wall was observed according to the subtraction and non-subtraction images to determine whether the stent adhered to the wall, and then 3D rotational angiography was performed to confirm whether the stent adhered to the wall. If the stent was still poorly attached, the Gateway™ balloon (Boston Sientific, America) with a matching stent size could be used to open the stent (9). Finally, conventional anteroposterior and lateral angiography of the whole brain was performed to determine whether there was any abnormality of intracranial vessels. Intracranial aneurysms with large volumes, daughter cysts, irregular shapes, or persistent jets could be filled with additional coils (10, 11).

A platelet function test was performed after routine oral administration of dual antiplatelet drugs (Aspirin 100 mg and Clopidogrel 75 mg) for 5 days before operation. If drug resistance occurred, ticagrelor was used instead (12, 13). Tirofiban (375 ug/h) was injected intravenously for 48 h after surgery and then changed to oral dual antiplatelet drugs for 6 months (an overlapping drug for 12 h to reduce the occurrence of ischemic events). If there were no ischemic symptoms or obvious intimal hyperplasia in the stent, it could be changed to long-term use of mono-antiplatelet drugs (aspirin 100 mg) (14).

### Aneurysm morphological metrics

As described in previous studies (15, 16), we calculated aneurysm morphological metrics—aneurysm maximum diameter (D_max_), aneurysm maximum height (H_max_), aneurysm vertical height (H), aneurysm middle diameter (D_middle_), aneurysm neck diameter (D_neck_), parent artery diameter (D_vessel_), size ratio (SR = H_max_/D_vessel_), aspect ratio (AR = H/D_neck_), bottle-neck ratio (BNR = D_middle_/D_neck_)—based on 3-dimensional angiographic reconstruction images.

### Postoperative evaluation and follow-up

The patients were followed up regularly after endovascular intervention. The modified Rankin Scale score (mRS) was used to evaluate the changes in clinical symptoms immediately after an operation and during the follow-up period. We defined mRS ≤ 2 as the good outcome and mRS > 2 as the poor outcome. Other safety indicators included symptomatic complications, technical complications, neointimal lining and stenosis, FD wall apposition, FD neck coverage (Classification A, B, C), and FD braid changes (Foreshortening, Fish mouthing, Brake bump deformation, Braid collapse) (16). DSA angiography was performed during the follow-up period. The results of cerebral angiography were evaluated by Consensus Grading Scale for Endovascular Aneurysm Occlusion (CGSFEAO) (17) immediately after endovascular intervention and during follow-up, Grade 0: Complete aneurysm occlusion; Grade 1: ≥ 90% aneurysm occlusion; Grade 2: 70–89% aneurysm occlusion; Grade 3: 50–69% aneurysm occlusion; Grade 4: 25–49% aneurysm occlusion; Grade 5: < 25% aneurysm occlusion.

### Statistical methods

All data were analyzed by SPSS 25.0 software. Shapiro-Wilk test was used to the normalcy of a distribution. X̄ ± s represented the continuous variables that conform to the normal distribution, and those that do not conform to the normal distribution were described by M (median) and IQR (interquartile range). Categorical variables are expressed by frequency and proportion. Univariate logistic regression analysis explored the key factors (related to aneurysm morphology, and treatment) of complete occlusion of tandem aneurysms. *p* < 0.05 was considered statistically significant.

## Results

### Baseline characteristics

A total of 38 aneurysms were included in 16 patients (6 male, 10 female). The mean age was 60.9 ± 11.2 years, ranging from 31 to 76 years. There were 12 cases of hypertension, 1 case of diabetes, and 3 cases of smokers. Ten cases (62.5%) presented with headache or dizziness, 3 cases (18.75%) were found by chance, and 3 cases (18.75%) had ischemic symptoms. There were 11 cases of 2 aneurysms, 4 instances of 3, and 1 case of 4. All aneurysms were located in the internal carotid artery, including 6 in the cavernous sinus segment, 21 in the ophthalmic segment, 5 in the posterior communicating segment, 3 in the Anterior choroidal segment, 1 in the paraclinoid segment, and 2 in the cervical segment. Thirty-five saccular and 3 fusiform aneurysms present 13 wide-neck, 25 relatively wide-neck, and 1 narrow-neck. Among them, 28 (73.7%) aneurysms maximum diameter was less than 5 mm, and 10 (26.3%) aneurysms had a maximum diameter of 5–15 mm (Table 1). The median value of D_max_, H_max_, H, D_neck_, D_middle_, SR, BNR were 4.27 (3.57–5.41)mm, 2.78 (1.78–4.427)mm, 2.53 (1.27–3.83)mm, 3.28 (2.77–4.88)mm, 3.27 (2.71–5.37)mm, 0.78 (0.50–1.07), 0.99 (0.985–1.03). The mean value of AR and D_vessel_ were 0.711 ± 0.37 and 3.62 ± 0.61 mm individually (Table 2).

**Table 1:** Demographic data and aneurysm characteristics of patients with tandem aneurysms treated with the FDD.

Case no.	Age (yrs)	Sex	Side	Location	Total no. of aneurysms	Aneurysm type				Aneurysm size (mm)				Treatment	No. of FDDs	FDD size (mm)
						1	2	3	4	1	2	3	4			
1	76	F	Rt	Ophthalmic, cavernous	2	S	S	—	—	7.1	6.4	—	—	PED (2×)	1	3.5 × 15
2	31	F	Lt	Cavernous, ophthalmic (2×)	3	S	S	S	—	8.4	4.4	3.9	—	PED (3×)	1	3.0 × 35
3	47	M	Lt	Ophthalmic (2×)	2	S	S	—	—	4.8	3.7	—	—	PED (2×)	1	4.0 × 20
4	65	M	Rt	Ophthalmic (2×), cavernous (1×), PCoA (1×)	4	S	S	S	S	5.4	3.5	3.2	2.8	PED (4×)	1	3.0 × 30
5	59	F	Rt	Ant choroidal, PCoA	2	S	Fu	—	—	4.3	3.6	—	—	PED (2×)	1	3.5 × 20
6	67	M	Lt	Ophthalmic (3×)	3	S	S	S	—	4.3	4.3	2.9	—	PED (3×)	1	4.25 × 25
7	65	M	Rt	Ant choroidal, ophthalmic	2	S	S	—	—	3.7	3.5	—	—	PED (2×)	1	4.0 × 30
8	60	F	Lt	Ophthalmic (2×), cavernous	3	S	S	S	—	4.8	3.7	3.7	—	PED (3×)	1	3.5 × 25
9	65	F	Rt	Ophthalmic (2×)	2	S	S	—	—	4.6	2.7	—	—	TFD (2×)	1	4.5 × 20
10	57	F	Lt	Ophthalmic, paraclinoid	2	Fu	S	—	—	6.7	4.6	—	—	Coil/TFD; TFD	1	4.5 × 25
11	56	F	Lt	Ant choroidal, PCoA	2	S	S	—	—	4.4	3.7	—	—	TFD (2×)	1	4.0 × 15
12	71	F	Rt	Cavernous (2×)	2	S	S	—	—	11.3	3.5	—	—	TFD (2×)	1	4.0 × 35
13	73	M	Rt	PCoA, ophthalmic (2×)	3	S	S	S	—	8.6	4.1	3.5	—	Coil/TFD; TFD (2×)	1	4.5 × 25
14	65	M	Lt	Ophthalmic (2×)	2	S	S	—	—	4.5	3.1	—	—	Coil/TFD; TFD	1	4.5 × 20
15	50	F	Lt	PCoA, ophthalmic	2	S	S	—	—	7.3	5.4	—	—	Coil/TFD (2×)	1	4.5 × 25
16	67	F	Lt	Cervical (2×)	2	Fu	S	—	—	7.3	4.6	—	—	Coil/TFD; TFD	1	5.0 × 25

—, not applicable; ant, anterior; F, Female; Fu, Fusiform; M, Male; PCoA, posterior communicating artery; S, saccular aneurysm.

**Table 2:** Aneurysm morphological metrics of patients with tandem aneurysms treated with the FDD.

Case no.	H_max_ (mm)	H (mm)	D_middle_ (mm)	D_neck_ (mm)	D_vessel_ (mm)	SR	AR	BNR
1	5.668	4.951	7.072	6.45	2.852	1.987	0.768	1.096
	5.805	5.548	6.428	3.988	3.665	1.584	1.391	1.584
2	3.127	2.875	3.838	3.882	2.942	1.063	0.741	0.989
	3.345	3.271	4.373	4.285	4.329	0.773	0.764	1.021
	5.438	4.64	8.424	8.44	2.977	1.827	0.55	0.998
3	3.872	3.664	4.803	4.479	4.600	0.842	0.818	1.072
	2.209	2.147	2.683	2.716	3.734	0.591	0.791	0.988
4	2.362	1.433	3.458	3.508	2.871	0.823	0.409	0.986
	2.076	1.301	3.138	3.168	2.482	0.836	0.411	0.991
	2.110	1.807	2.774	2.772	2.568	0.822	0.652	1.001
	3.380	2.478	5.423	5.450	3.646	0.927	0.455	0.995
5	4.277	4.126	4.015	3.726	3.188	1.342	1.108	1.078
	1.311	0.679	2.24	2.271	3.59	0.365	0.299	0.986
6	1.367	1.161	2.323	2.359	4.259	0.321	0.492	0.985
	2.595	2.492	3.174	3.223	4.283	0.606	0.773	0.985
	2.813	2.709	2.807	2.907	4.081	0.689	0.932	0.965
7	2.713	2.568	2.911	2.982	3.489	0.778	0.861	0.976
	2.964	2.678	3.315	3.263	3.728	0.795	0.821	1.016
8	2.739	2.109	4.777	4.824	3.66	0.748	0.437	0.99
	1.721	1.099	2.929	2.974	3.739	0.46	0.37	0.985
	1.473	0.864	2.421	2.448	3.739	0.394	0.353	0.989
9	2.131	1.348	2.717	2.758	2.765	0.771	0.489	0.985
	3.657	3.381	4.634	4.646	3.527	1.037	0.728	0.997
10	5.54	4.808	6.66	6.703	3.599	1.539	0.717	0.994
	2.996	2.927	3.223	3.302	4.436	0.675	0.887	0.976
11	1.358	0.967	2.06	2.088	3.704	0.367	0.463	0.987
	1.354	0.675	2.233	2.267	3.465	0.391	0.298	0.985
12	9.533	9.174	11.256	9.551	3.201	2.978	0.96	1.179
	2.45	2.184	2.667	1.048	3.494	0.701	2.083	2.544
13	7.851	7.647	8.629	6.67	3.561	2.205	1.146	1.294
	1.796	0.857	3.03	3.072	4.115	0.436	0.279	0.986
	1.344	1.038	2.326	2.367	4.143	0.324	0.439	0.983
14	1.602	1.438	2.766	2.818	3.109	0.515	0.51	0.981
	4.543	3.742	4.006	3.839	4.17	1.089	0.975	1.044
15	7.237	6.974	7.268	5.040	2.333	3.101	1.384	1.442
	4.377	4.080	5.351	5.399	4.208	1.010	0.756	0.991
16	3.59	2.83	5.82	7.28	4.61	0.779	0.389	0.799
	1.41	1.06	2.33	3.18	4.61	0.306	0.333	0.89
Mean ± SD/M, IQR	2.78 (1.78–4.427)	2.53 (1.27–3.83)	3.27 (2.71–5.37)	3.28 (2.77–4.88)	3.62 ± 0.61	0.78 (0.50–1.07)	0.711 ± 0.37	0.99 (0.985–1.03)

AR, aspect ratio; SD, Standard deviation; D_vessel_, parent artery diameter; BNR, bottle-neck ratio; M, median; IQR, interquartile range; SR, size ratio; D_neck_, aneurysm neck diameter; D_middle_, aneurysm middle diameter; H, aneurysm vertical height; H_max_, aneurysm maximum height.

### Angiographic and clinical outcome

One FDD stent was implanted in all patients. A total of 16 FDD stents were implanted in 38 aneurysms, including 8 PED stents and 8 TFD stents. The technical success rate was 100%. Thirty-two aneurysms were treated with simple FDD stent implantation, and 6 aneurysms were treated with FDD stent combined with coil embolization. Immediately after surgery, DSA angiography showed that the parent artery and branch vessels were unobstructed, and the stents were well adhered to the wall. There were 31 CGSFEAO grade 5 (81.6%), 2 CGSFEAO grade 4 (5.2%), 2 CGSFEAO grade 1 (5.2%) and 3 CGSFEAO grade 0 (8%). The complete occlusion rate of the study was reached 8%. Immediate postoperative mRS of all patients had less than 2 (Table 3).

**Table 3:** Outcomes in patients with tandem aneurysms treated with the FDD.

Case no.	Total no. of aneurysms	CGSFEAO grading post-deployment for aneurysms A–D, as applicable				CGSFEAO grading at last FU for aneurysms A–D, as applicable				Last DSA— FU (Months)	Complications
		A	B	C	D	A	B	C	D		
1	2	A	A	—	—	D	D	—	—	37	None
2	3	A	A	A	—	D	D	D	—	13	None
3	2	A	A	—	—	D	D	—	—	3	None
4	4	A	A	A	A	D	D	D	D	6	None
5	2	A	A	—	—	D	D	—	—	24	None
6	3	A	A	A	—	D	A	D	—	12	None
7	2	A	A	—	—	D	D	—	—	12	None
8	3	A	A	A	—	A	A	D	—	4	None
9	2	A	A	—	—	B	D	—	—	34	Intraop vasospasm with thrombosis
10	2	A	A	—	—	D	D	—	—	4	None
11	2	A	A	—	—	B	D	—	—	17	None
12	2	A	A	—	—	A	D	—	—	29	Vascular dissection
13	3	A	A	A	—	D	A	D	—	27	None
14	2	D	B	—	—	D	D	—	—	3	None
15	2	D	C	—	—	D	D	—	—	6	None
16	2	D	C	—	—	D	D	—	—	4	None

FU, follow-up; intraop, intraoperative; CGSFEAO, Consensus Grading Scale for Endovascular Aneurysm Occlusion.

**Figure 1:**
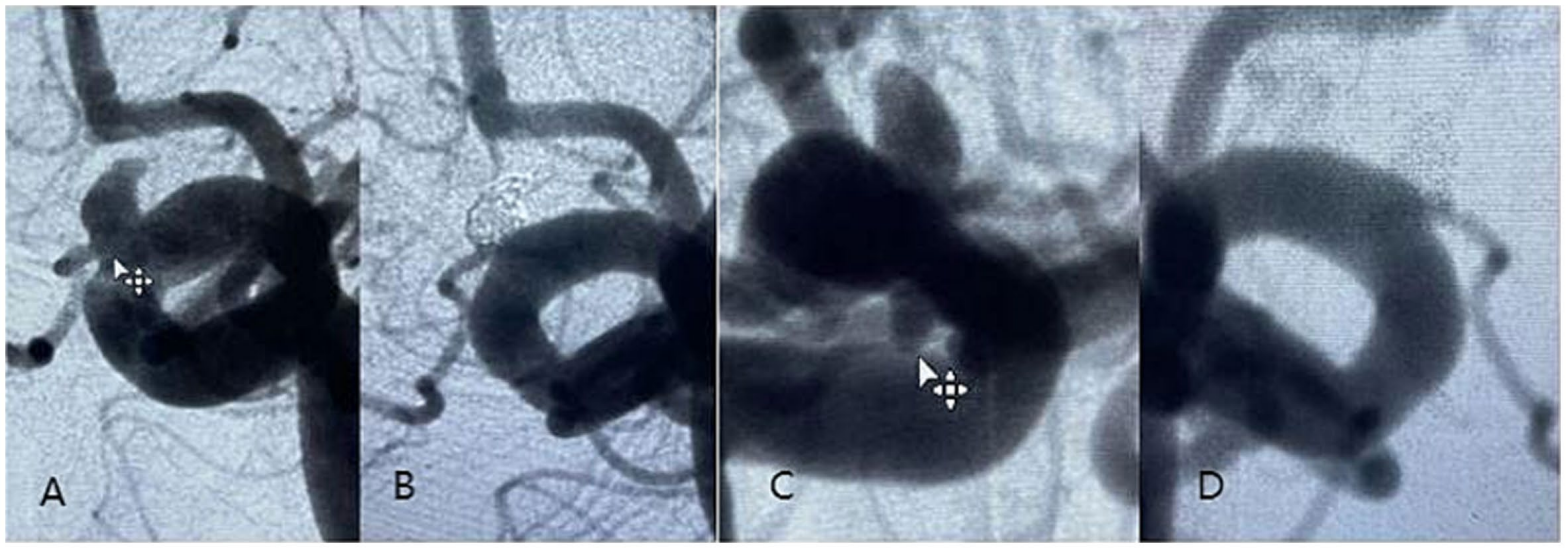
An elderly male patient with a small left mirror-image carotid-ophthalmic aneurysm was treated with a 4.5–20 mm Tubridge, in which the anterior wall aneurysm was filled with additional coils. **(A)** Preoperative DSA angiogram of anterior wall aneurysm showed that the ophthalmic artery emanated from the neck of the aneurysm; **(B)** DSA angiogram at 6 months follow-up showed complete occlusion of anterior wall aneurysms; **(C)** Preoperative DSA angiogram showing posterior wall aneurysm; **(D)** DSA angiogram at 6 months follow-up showed complete occlusion of posterior wall aneurysms.

**Figure 2:**
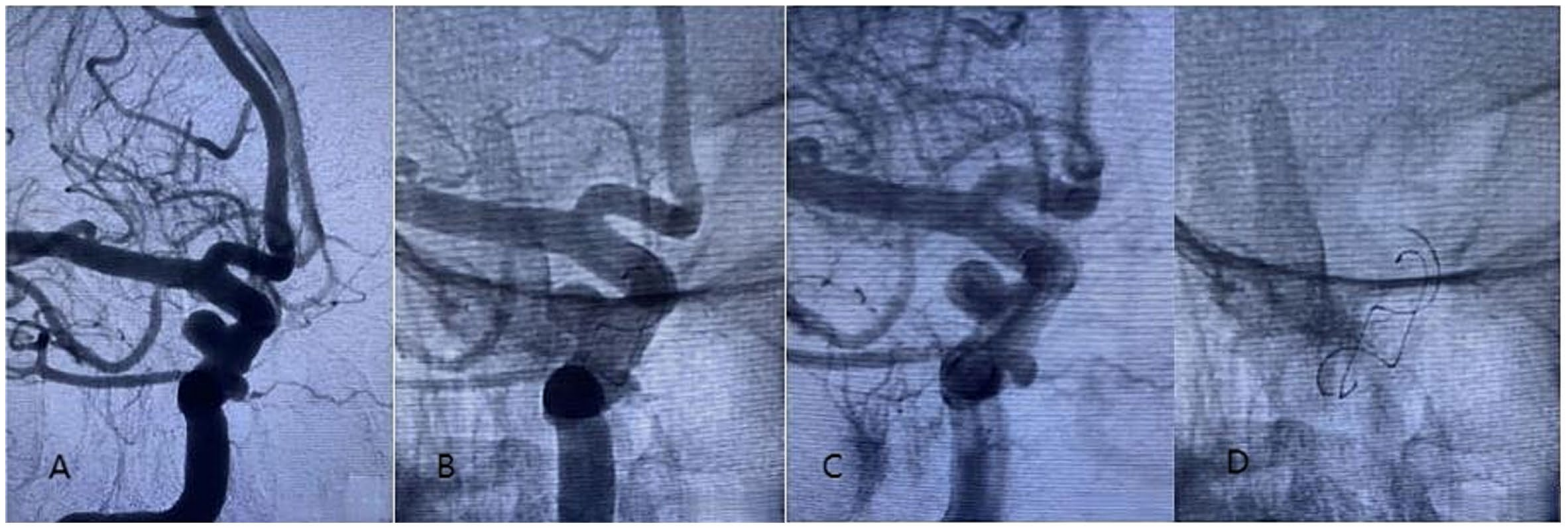
A 65-year-old woman diagnosed with tandem aneurysms in the right internal carotid cavernous segment was treated with a 4.5-20 mm Tubridge. **(A–D)** Stents in different phases.

All patients were followed up clinically for a median of 25.5 months (15.5, 28.7 months). At the last follow-up, no patient had died and no new neurological deficit was found in the responsible artery territory. All patients had good clinical outcomes. DSA angiography was performed in all patients to evaluate the occlusion of cerebral aneurysms during the follow-up period. Its median follow-up time was 14 months (6, 27 months). Of the 38 aneurysms, there were 5 CGSFEAO grade 5 (13.2%), 1 CGSFEAO grade 4 (2.6%), 1 CGSFEAO grade 2 (2.6%) and 31 CGSFEAO grade 0 (81.6%) (Table 3). The complete occlusion rate of cerebral aneurysm in this study was 81.6%, including 85.7% in the PED group and 76.5% in the TFD group, and there was no significant statistical difference between the two groups. A typical case of complete occlusion is shown in Figure 1. A case of Stents in different phases is shown in Figure 2.

### Complication

**Figure 3:**
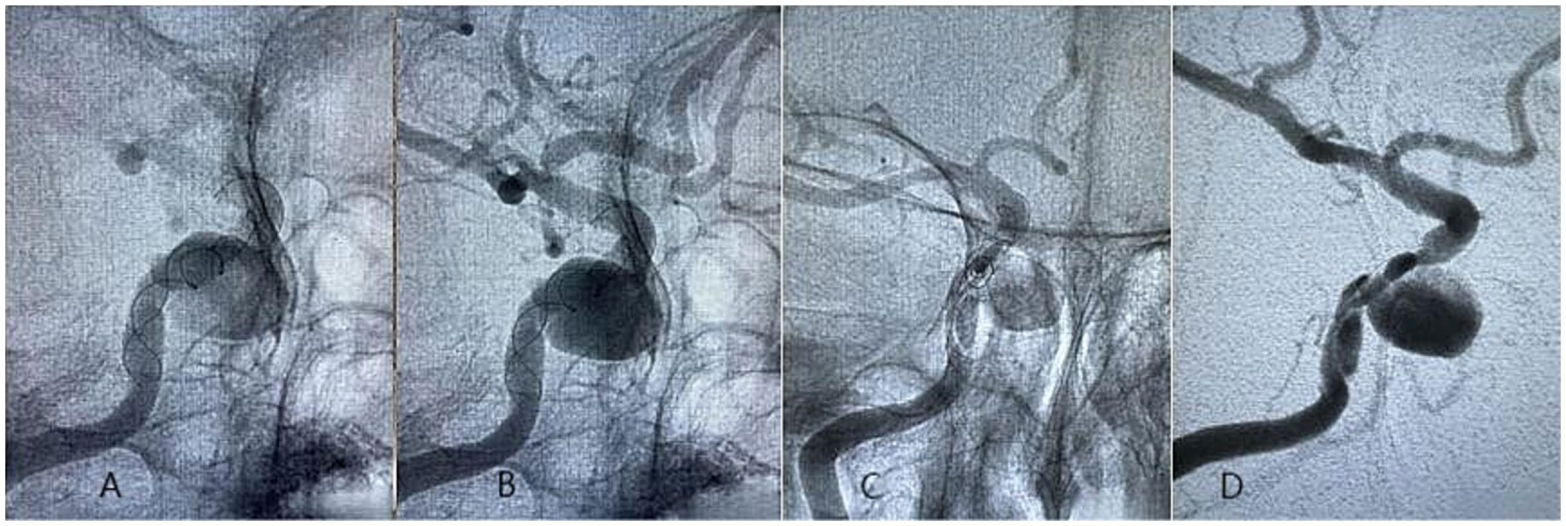
A 70-year-old woman diagnosed with tandem aneurysms in the right internal carotid cavernous segment was treated with a 4.0-35 mm Tubridge. **(A,B)** Postoperative DSA angiogram showed no clear vascular abnormalities; **(C,D)** At 27 months of follow-up, DSA imaging showed the formation of vascular dissection, and compressing the stent to deformation and collapse.

There were no deaths in the present study. Three patients were found with neointimal lining in a stent (< 50%) without obvious stenosis, with 1 treated by TFD and 2 treated by PED. The TFD covered 11 branches without significant narrowing and occlusion. The PED covered 9 branches, with 2 mild stenoses (< 50%) and 2 occlusions, all asymptomatic. FD wall apposition is well in all patients. Three patients treated by TFD involved branch vessel, including 1 patient with full neck coverage (Classification C) and 2 patients with partial neck coverage (Classification B). Two cases incorporating branch vessel in PED group are both Classification B. One patient occurred braid collapse in TFD group. FD braid changes were not found in PED group. One complication occurred intraoperatively, and another was found during the follow-up period. Case 1 had intraoperative vasospasm with thrombosis at the neck of the aneurysm. Tirofiban 500ug and 5 mg fasudil were immediately infused through the catheter. Thrombolysis and arterio-spasm elimination were observed after 5–10 min. The final mRS of the patient was 0. Case 2 angiography at follow-up showed that the end of the TFD was compressed, and vascular dissection was formed outside the stent. The operator carefully considered that the proximal end of the stent was located in the transitional zone between the diseased blood vessels and normal blood vessels, and the proximal end of the stent caused intimal injury with vascular peristalsis, thus forming vascular dissection, and compressing the stent to deformation and collapse (Figure 3). Likewise, the mRS of the patient was 0 (Table 3).

### Univariate logistic regression analysis

It showed that Age, Gender, D_max_, H_max_, H, D_middle_, D_neck_, D_vessel_, AR, SR, BNR, location, involvement of branch vessels, combination of coils embolization, and type of FDD were not clinical factors affecting the complete occlusion of tandem aneurysm (Table 4). Interestingly, the results of this study suggest that smaller AR (complete occlusion group 0.75 Vs. incomplete occlusion group 0.54, *p* = 0.171) and anterior wall aneurysms (complete occlusion group 38.7% Vs. incomplete occlusion group 71.4%, *p* = 0.132) tend to incomplete occlusion.

**Table 4:** Related factors affecting complete occlusion of intracranial tandem aneurysms treated with FDD.

Clinical factors	Complete occlusion (*n* = 31)	Incomplete occlusion (*n* = 7)	Univariate logistic regression		
			OR value	95%CI	*p* value
Age, (Mean, SD), year	59.9 ± 12.23	64.5 ± 6.24	1.048	0.952–1.153	0.336
Gender, (Femal, *n*)	17	5	0.486	0.081–2.897	0.428
AR, (Mean, SD)	0.75 ± 0.39	0.54 ± 0.24	0.086	0.003–2.881	0.171
D_vessel_, (Mean, SD), mm	3.61 ± 0.64	3.64 ± 0.51	1.071	0.275–4.18	0.921
BNR, (M, IQR)	0.991 (0.985–1.044)	0.986 (0.985–0.99)	0.175	0–70.576	0.175
SR, (M, IQR)	0.82 (0.59, 1.09)	0.61 (0.44, 77)	0.84	0.23–3.13	0.797
D_Max_, (M, IQR), mm	4.28 (3.51, 5.45)	4.12 (3.70, 4.82)	1.06	0.69–1.62	0.8
D_neck_, (M, IQR), mm	3.51 (2.77, 5.04)	3.07 (2.76, 4.82)	1.024	0.66–1.59	0.916
D_middle_, (M, IQR), mm	3.46 (2.70–5.42)	3.03 (2.72–4.78)	1.016	0.694–1.488	0.935
H, (M, IQR), mm	2.71 (1.43–4.08)	1.35 (0.97–2.49)	0.899	0.568–1.421	0.648
H_max_, (M, IQR), mm	3.13 (2.08–4.54)	2.13 (1.72–2.74)	0.933	0.598–1.456	0.761
Anterior wall aneurysm (*n*)	12	5	0.253	0.042–1.516	0.132
Involving branching vessel (*n*)	5	1	1.15	0.11–11.78	0.904
Combined coils emboliza-tion (*n*)	6	0	0	0	0.999
Implanting PED (*n*)	18	3	1.85	0.35–9.69	0.469

AR, aspect ratio; SD, Standard deviation; θ_F_, aneurysm inflow angle; deg, degree; θ_A_, aneurysm inclination angle; D_vessel_, parent artery diameter; BNR, bottle-neck ratio; M, median; IQR, interquartile range; SR, size ratio; D_max_, aneurysm maximum diameter; D_neck_, aneurysm neck diameter; D_middle_, aneurysm middle diameter; H, aneurysm vertical height; H_max_, aneurysm maximum height.

## Discussion

In patients with intracranial aneurysms, multiple aneurysms are one of the indications for treatment, accounting for 14%–34% (1). Compared to other multiple aneurysms, tandem aneurysms are distinguished by their unique anatomical location. Firstly, tandem aneurysms mostly small in size are commonly found in the supraclinoid segment of the ICA, which increases the difficulty of traditional treatment. Secondly, tandem aneurysms are often accompanied by abnormalities in the vascular wall of the segment, meaning that even if the tandem aneurysm is cured, there may still be new aneurysms due to segmental vascular wall abnormalities (1, 2, 18). Therefore, such lesions often require active intervention. FDD is a blood flow remodeling device developed based on hemodynamic studies of intracranial aneurysms. Its emergence has changed the concept of endovascular treatment of intracranial aneurysms and shifted the previous intracapsular embolization to the reconstruction of the parent artery (19). The FDD is the well-suited treatment for such lesions, which can cure aneurysms and repair abnormal blood vessel walls. On the other hand, the operation of traditional intravascular interventional therapy is reduced, and the safety of the operation is improved.

Previous studies have shown that the complete occlusion rate of FDD in the treatment of tandem aneurysms is about 75.9–94.1% (1, 2, 7, 20, 21), which is consistent with the results of this study. Awad et al. (7) analyzed the clinical efficacy of PED treating intracranial tandem aneurysms. A total of 38 aneurysms were included in 17 patients. Aneurysms were mostly located in the supraclinoid segment of the internal carotid artery (32/38, 84.2%), and the complete occlusion rate of aneurysms was 79%. Feng et al. (1) compared PED with coil embolization in the treatment of intracranial tandem aneurysms, the results showed that the complete occlusion rate of the PED group was significantly better than that of the coil embolization group (94.1% Vs.77.4, *p* = 0.038); the proportion of patients with mRs 0–2 points in the PED group reached 96%, which was not statistically different from that in the coil embolization group (90.7%). Lin et al. (8) compared FD with conventional stent-assisted coil embolization of intracranial tandem aneurysms. Among them, six patients (12 aneurysms) received FDD treatment and seven patients (11 aneurysms) received stent-assisted coil embolization. In the FD group, 5 patients (10 aneurysms) were followed up by DSA, and 9 aneurysms were completely occluded. No adverse events occurred during the perioperative period and follow-up period. All patients in the coil embolization group were followed up by DSA, eight aneurysms were completely occluded, and 1 case of ischemic complications occurred during the follow-up period. In this study, although the aneurysm occlusion rate was higher in patients receiving FD treatment, there was no significant difference between the FD group and the embolization group (*p* = 0.08). In addition, an important finding of this study was that the efficacy and safety of TFD in the treatment of tandem aneurysms in the internal carotid artery are similar to those of PED, which is consistent with previous research results. Cai et al. (20) conducted a multicenter retrospective clinical study on PED and TFD in the treatment of intracranial wide-neck aneurysms. The results showed that the complete occlusion rate of aneurysms (77.42 vs. 85.71%, *p* > 0.05) and safety (2.56 vs. 3.77%, *p* > 0.05) in the PED and TFD were comparable.

At present, the incidence of perioperative adverse events in the study of FD in the treatment of intracranial tandem aneurysms is 4.3%–15.4% (1, 2, 7, 8, 20, 21). Bhogal et al. (2) conducted a single-center retrospective study on the treatment of 169 intracranial aneurysms in 69 patients with FDD. The results showed that there were 3 cases of symptomatic complications (4.3%), 1 case of hematoma at the puncture site, 1 case of secondary nasal bleeding, and the last case of internal carotid artery occlusion due to discontinuation of antiplatelet drugs, resulting in large-area cerebral infarction and eventual death. The above-mentioned Awad et al. ‘s study (1) on intracranial tandem aneurysms received by PED found 2 cases of perioperative asymptomatic complications (11.8%), 1 case of carotid artery dissection, and 1 case of hemorrhage during auxiliary coil packing, both of which had no obvious postoperative symptoms. Johns et al. applied PED to treat 47 aneurysms in 20 patients (19). All aneurysms were located in the internal carotid artery, and 93.6% (44/47) were located in the supraclinoid segment of ICA. No complications occurred during the operation. Perioperative complications occurred in 3 cases (15%), including 2 patients with speech dysfunction and 1 patient with limb weakness. All 3 patients recovered well, and no neurological deficit was found in long-term follow-up.

The safety results of FDD in the treatment of intracranial tandem aneurysms in this study are similar to previous studies. There was 1 case of ischemic complication during the perioperative period. The patient had vasospasm and thrombosis at the neck of the tumor during the operation. The patient recovered well after transcatheter arterial infusion of tirofiban and fasudil, and the mRS was 0 at the last follow-up. During the follow-up period, one case of carotid artery dissection was found. The reasons for the surgeon’s inference were as follows: Firstly, the low tension release of TFD caused stent retraction to induce dissection at the proximal end of the parent artery; Secondly, the aneurysm itself was a dissecting aneurysm, but TFD did not completely cover the dissection segment; Thirdly, the proximal end of the stent was located in the transitional zone between the diseased blood vessels and normal blood vessels, and the proximal end of the stent caused intimal injury with vascular peristalsis, thus forming vascular dissection, and compressing the stent to deformation and collapse. The operator seriously considered the Third option as the most likely. It was recommended that patients undergo balloon dilatation before implantation of a flow diverter (enhanced flow diverter) or a conventional stent (fixed support to avoid displacement or further collapse of the first stent). Unfortunately, considering the absence of any symptoms and the high economic burden, the patient chose to continue the follow-up observation. At present, dual antiplatelet (Aspirin 100 mg daily + clopidogrel 75 mg daily) and lipid-lowering (atorvastatin 20 mg daily) drugs were continued without endovascular intervention. And the mRS was 0 at the last follow-up.

Previous studies have found that the morphology, location, size, involvement of branches, and distance between tandem aneurysms were related to the complete occlusions of cerebral aneurysms after FDD (21), but the most important factor is hemodynamic changes (12, 22). Mut et al. (22) established a hemodynamic model of intracranial tandem aneurysms treated by FDD. They found that compared with convex aneurysms, the blood flow inflow, average flow velocity, shear rate, and wall shear stress of the concave aneurysms of the parent artery after implanting FDD stents were significantly reduced, and the occlusion speed was faster. This study found no risk factors affecting the complete occlusion of anterior circulation tandem aneurysms treated by FDD due to the small sample size. Interestingly, the results of this study suggest that smaller AR and anterior wall aneurysms tend to incomplete occlusion which might be related with wide neck and more large flow impingement. If the sample size is large enough, it is possible to get encouraging results. The author believes that FD combined with loose coil packing in the treatment of anterior wall aneurysms may promote thrombosis in aneurysms, thereby improving the occlusion rate of aneurysms.

Finally, the authors believed that fusiform or saccular aneurysms of the internal carotid artery communicating segment, ophthalmic artery segment, clinoid segment, and cavernous sinus segment were prone to flow diverter implantation, and aneurysms of communicating segment and ophthalmic artery segment could also be surgically clipped theoretically. Our center preferred flow diverter intervention, which was relatively simple to operate, with low risk and few complications. Additional loose coiling with coils was feasible for aneurysms with large diameters (> 5 mm), with daughter cysts, with irregular shapes, with persistent jets, in the anterior wall of the internal carotid artery, or involving the ophthalmic artery or posterior communicating artery.

### Limitations

(1) This study was a small sample retrospective study; (2) Patients from the two centers were included in this study, and there may be differences in physician preferences; (3) This study only included anterior intracranial aneurysms, which was limited in representativeness.

## Conclusion

FDD is safe and effective in the treatment of tandem aneurysms in the internal carotid artery with a high occlusion rate and few complications. TFD is comparable to PED in the treatment of intracranial tandem aneurysms.

## Data Availability

The raw data supporting the conclusions of this article will be made available by the authors, without undue reservation.
